# Common intrusion factors and improvement measures based on case study of privacy impact assessment

**DOI:** 10.1371/journal.pone.0328180

**Published:** 2025-08-25

**Authors:** Jae-sik Yi, Dong-Seok Jang, Youn-Sik Hong

**Affiliations:** 1 Department of Computer Science and Engineering, Incheon National University, Incheon, Korea; 2 National Institute of Biological Resources, Korea; Lucian Blaga University of Sibiu: Universitatea Lucian Blaga din Sibiu, ROMANIA

## Abstract

As the Internet becomes increasingly widespread, various cybercrimes involving privacy, such as misuse, abuse, and leakage of privacy in integrated information systems that contain privacy data, are increasing worldwide. To address such intrusions, privacy impact assessments (PIAs) of information systems have been performed. Various studies, including PIAs, have been conducted to establish PIA frameworks (PIAF), surveys, and analyses to investigate intrusion cases. Impact assessments based on assessment items and low-level PIAs that analyze intrusion factors differ among countries. The PIAF in the Netherlands comprises three stages, that in Canada comprises four stages, and that in Korea comprises three stages, each of which defines subprocesses. In this study, the PIAFs of these countries were investigated and compared. We also compared the assessment items of ISO/IEC 27701 and Korea’s PIAF. We analyzed PIAs conducted on information systems operated by public institutions in Korea. Through the analysis of these PIAs, we derived the factors causing intrusions in Korea and proposed improvement points for each intrusion factor. We expect that these analysis results can be effectively applied to other countries.

## 1. Introduction

Various information systems that contains privacy data are in use in both the public and private sector around the world. In recent years, privacy protection has become a key issue in information systems, along with operational efficiency and customized services. Information systems are becoming increasingly vulnerable to privacy violations and abuses by both external and internal hackers and staff. This has become a social problem as many people have been victimized by various cybercrimes, including voice phishing, using leaked privacy.

Movements to securely handle privacy in information systems have been initiated in Australia and Canada. The concept of privacy was defined as a human right in the European Union (EU) Charter of Fundamental Rights in 2000. Subsequently, its importance was recognized globally, leading to the emergence of an innovative method called privacy impact assessment (PIA) [1].

Since the mid-1990s, the concept and identification of PIAs have evolved from technical assessment, impact statement, and impact assessment to PIAs. Definitions of PIAs began to appear in legislation, policy, and guidance materials. During this process, Australia’s Roger Clarke played a leading role. In Australia, a PIA is an assessment of the actual or potential impacts on privacy and how they can be mitigated [1].

PIAs are guided by five principles: continuity of assessment, adaptability, protection of individual privacy, organizational accountability, and transparency. These ensure that privacy risks are proactively managed across evolving systems [2].

Research on frameworks for implementing more realistic PIAs has continued. The recommendations for the PIA framework (PIAF) for the EU are divided into two sets. The first set analyzes why and how to introduce a PIA policy, identifies and describes the components of a PIA, and discusses the role of data protection authorities (DPAs) in the PIA process, addressed by policymakers. The second set is addressed by assessors who perform PIAs and for whom guidance on best practices is provided [2].

In Korea, the Privacy Protection Act came into effect on September 30, 2011. PIAs in Korea are defined as assessments to analyze risk factors and derive improvement measures when there is concern about privacy intrusion when handling privacy files. The Ministry of Government Administration and Home Affairs published a guide for performing a PIA to prevent privacy intrusion incidents in advance when conducting business involving the processing of privacy [3–4].

The PIA Framework (PIAF) in Korea is structured into three stages: preparation, execution, and implementation. These stages encompass planning and budgeting, assessment of privacy risks and flows, and verification of whether mitigation measures were applied. The structured approach ensures that privacy is embedded into the entire system development lifecycle in the public sector [5].

This study has two objectives. The first objective is to safely handle privacy when operating an information system that involves the processing of privacy. The second objective is to prevent large-scale privacy leakage incidents due to violations of the rights of information subjects or various cyberattacks from inside and outside the organization. First, we analyzed PIAFs in foreign countries and compared them with those in Korea even though it is difficult to compare detailed items because of the lack of data from foreign countries. In addition, we derived key items that can be used as references in the actual field by focusing on specific cases. For this purpose, we analyzed five PIA projects that were executed during the information system design and analysis phases in Korean public institutions. In this process, we introduced in detail the 4 areas and 18 fields applied to PIAFs in Korea. In addition, we present the results of evaluating the target information system by applying assessment items consisting of 4 areas and 18 fields. Based on the assessment results, we derived 13 items that should be considered in the PIA project based on the frequency of partially implemented or non-implemented items. For each of the 13 items, privacy intrusion factors are derived, and improvement measures are proposed for each factor.

## 2. Related works

David Tancock, et al. [1] explored the development of PIAs and systematic processes for evaluating the potential privacy impacts of projects or proposals. Emerging from concepts such as technology assessments and environmental impact statements, PIAs have gained prominence across jurisdictions, including Canada, New Zealand, the US, Australia, and Europe. They identified and mitigated privacy risks, offering transparency, stakeholder engagement, and risk assessments. Despite varied applications across public and private sectors, PIAs are increasingly important given automated decision-support systems and cross-border data processing.

David Wright, et al. [2] analyzed PIA methodologies across seven jurisdictions, including Australia, Canada, Hong Kong, Ireland, New Zealand, the United Kingdom, and the United States. They highlighted the value of PIAs in identifying and mitigating privacy risks, offering comparative insights into each jurisdiction’s legal frameworks, stakeholder engagement, and best practices. Case studies and expert analyses reveal key criteria for effective PIAs, such as transparency, public consultation, and comprehensive risk assessment. They concluded with recommendations for a unified PIAF in Europe, emphasizing mandatory assessments for high-risk projects, public consultation, and alignment with the EU Data Protection Directive to strengthen data protection and privacy rights.

Paul De Hert, et al. [6] provided a comprehensive framework for PIAs. They reviewed PIA practices across seven jurisdictions, including Canada, the US, and Australia, to identify effective elements that can be adapted for the EU. They provided two sets of recommendations: one for policymakers advocating for high level support and compulsory PIAs; another for assessors, emphasizing best practices such as transparency, stakeholder consultation, and risk management. Their framework advocates for a consistent, scalable, and accountable PIA policy in the EU, aligned with the General Data Protection Regulation (GDPR), to safeguard data privacy rights.

Konstantina Vemou, et al. [7] also presented a comprehensive framework for PIA methods to help organizations select the most suitable approach. They analyzed 9 prominent PIA methods, including those developed by DPAs and academic researchers, against 17 criteria such as risk assessment guidance, automation tools, and stakeholder consultation. Their assessment revealed significant variations among methods, with gaps such as insufficient guidance on team selection and a lack of automated tools. The authors recommended a more holistic approach that addresses both individual and organizational risks, provides comprehensive risk assessment metrics, and includes practical privacy controls and clear role assignments, ultimately aiming to improve PIA practices and align them with privacy-by-design principles.

Jeroen van Puijenbroek, et al. [8] investigated the practical application of PIAs across 14 Dutch organizations. Despite no legal requirement at the time, most organizations conducted PIAs assuming they were mandatory, focusing mainly on compliance rather than genuinely protecting data subjects. They revealed a predominantly organization-centric approach to privacy risks, limited stakeholder consultation, and vague risk assessment methodologies. Consequently, PIAs typically address the effects rather than the causes of privacy risks, leading to compliance but not necessarily privacy-friendly systems. They advocated for a more rigorous and transparent PIA process that prioritized data subjects and integrated privacy-by-design principles to develop systems that aligned with both regulatory requirements and societal norms.

David Wright, et al. [9,10] examined PIA policies and methodologies in Australia, Canada, Ireland, New Zealand, the UK, and the US. They identified similarities and differences in PIA approaches, with each country displaying strengths and shortcomings. The analysis used 18 benchmarks, including whether PIAs are mandatory, the involvement of stakeholders, publication of PIA reports, and risk management strategies. They argue that the EU should leverage these international experiences to construct a unified and optimized PIAF as part of its proposed Data Protection Regulation. The authors recommended incorporating the best elements, such as transparency, stakeholder engagement, and independent audits, to ensure effective privacy risk management across EU Member States.

Reuben Binns [11] explored how data protection impact assessments (DPIAs) have evolved from voluntary tools into mandatory requirements under the EU GDPR. He argues that the GDPR has transformed DPIAs into a form of “meta-regulation,” where organizations are held accountable for self-regulating their privacy practices. DPIAs aim to leverage the internal management structures of organizations to identify, assess, and mitigate data privacy risks while providing flexibility for context-specific solutions. Despite concerns about regulatory burden and vague risk definitions, he emphasized that independent and stakeholder scrutiny, stability, and support are critical to effective meta-regulation. Ultimately, he advocated for DPIAs as a promising regulatory tool that, if effectively implemented, can improve privacy risk management and compliance in the EU.

Konstantina Vemou, et al. [12] provided practical guidance for implementing PIAs. They proposed a comprehensive PIA process that incorporates best practices from existing guidelines and privacy research, along with an assessment framework comprising 17 criteria. This framework was applied to assess nine commonly used PIA methods, revealing gaps in practical guidance and risk assessment. They highlighted the importance of a systematic approach, including threshold analysis, stakeholder consultation, and practical risk assessment metrics, while advocating for a privacy-by-design approach that eliminates risks rather than mitigating them. Their proposed PIA process is intended to help organizations effectively organize and implement PIA projects, ensuring compliance with the GDPR and fostering a culture of privacy protection.

While Wright et al. provided theoretical frameworks for PIA, they lacked practical case-based validation. Vemou et al.’s assessment criteria were comprehensive, but no real-world risk factors were examined. Our study bridges this gap by identifying intrusion-prone items and proposing actionable improvements derived from live PIA reports.

Marie Shroff [13] provided a detailed practical guide for preparing a PIA report.

In Korea, PIAs are implemented according to the guidelines and manuals provided by designated assessment institutions [3–4]. Jeon Dong-jin et al. [14–15] conducted a PIA on systems such as the Medical Order Communication System, Picture Archiving and Communication System, and customer management systems of small shopping malls. They assessed the personal information management systems (PIMSs) of the target institutions and systems and items in the privacy lifecycle and specific IT technology application fields based on 4 assessment areas and 15 assessment items. Shin Sang-kyu et al. [16–17] performed a PIA on the electronic chart system of Saitama, Japan. They developed and conducted an assessment method comprising a conformity assessment for risk mitigation plans and a validity assessment for requirements.

While prior works such as Wright et al. and Vemou et al. offer comprehensive overviews and benchmarks of PIA frameworks, they rarely provide case-driven analyses with specific technical risk factors or operational improvement strategies. Our work addresses this gap by identifying intrusion factors from real PIA execution results and proposing item-level countermeasures.

Recent studies have explored diverse approaches to privacy protection and secure authentication. For example, Sahar Altalhi, et al. [18] proposed an efficient offline signature verification method that can be applicable in access control for secure document signing. Similarly, Adnan Gutub [19] discussed personalized secret-sharing strategies to enhance the reliability of privacy-preserving authentication systems. Research in [20] used deep learning to analyze emotional privacy signals in large-scale public events, suggesting the importance of real-time affective data processing in privacy risk assessments. Recent literature also addresses privacy-protective steganography [21–22] and smart device data protection [23], while studies like [24] underscore the role of institutional motivation and user trust in adopting secure IT systems.

Aditya Kumar Sahu, et al. [22] addressed that the hospitality industry has in the recent past been targeted by cybercrimes and management of cybercrimes are divided into facets like policies of security frameworks, cyber-threats, and management appreciating the value of Information Technology investment. There study’s purpose is to examine cybersecurity activities of network threats, electronic information, and the techniques of preventing cybercrime in hotels. Sara Manour Almutairi, et al. [24] addressed that the work evaluated using IT considering particular concerns within the Saudi region. It suggests the possible tuning strategies making teachers motivated to adopt IT in their educational job. The research surveys 100 different schools to identify the impact of using IT and surveys are build utilizing different paragraphs specifically designed according to the region situations as well as group categorization, that is of students, teachers, principals and parents. The work used means and standard deviations to analyze the results and build specific recommendations geared towards teachers’ motivation. The study proved specific urgency for improvement tuning need within the schooling system. Its main advises are found urgently essential to be considered in order to motivate the teachers via specific strategies leading to interesting educational improvements.

From this literature review, we could not find any studies that specifically identified intrusion factors based on PIA results or proposed improvement measures for each factor. Although previous studies presented an overview, methodology, and framework for PIAs at a conceptual level, it was difficult to find specific cases where these concepts were applied and evaluated in practice.

## 3. PIA procedures and comparison

### 3.1. Overview of privacy

#### 3.1.1. *Concept of privacy in Europe.*

The concept of privacy has been recognized in different regions and cultures around the world. For over a century, philosophers, lawyers, and political theorists have sought to understand the dimensions of privacy from various perspectives. However, attempts to define a unified definition of privacy have been controversial. Some definitions have been criticized for not sufficiently distinguishing privacy from the other four concepts: privacy as a human right, social needs, interpretation of privacy as privacy, and PIA. This is because privacy has many meanings in various contexts, as summarized below [1].

- Philosophical perspective: In Europe, people are considered essential in themselves. The concepts of “human dignity” and integrity play an important role in some countries, as do the concepts of individual autonomy and self-determination.
- Psychological perspective: People need private personal space, and this applies not only in public places but also behind closed doors and curtains. Personal space is the area surrounding an individual that he or she psychologically regards as his or her own; people enjoy and want to protect their private space; privacy is an individual’s concern to maintain a “personal space” free from interference from other people or organizations.
- Sociological perspective: People should be able to act freely and socialize with others, but there should be no constant threats.
- Economic perspective: People should be free to innovate, and every innovator is “different from the norms of the time” and may recognize that he or she could be at risk if he or she lacks personal space to experiment.
- Political perspective: People should be able to think, argue, and act freely. Some types of surveillance in public places or work environments can restrict human behavior and speech [1].

#### 3.1.2. *Definition of privacy-related terms.*

Privacy-related terms are defined in Table 1 on the basis of Article 2 (Definition) of Korea’s Privacy Protection Act [3] and the Notice on PIA [4].

**Table 1:** Definition of privacy-related terms [3,5,25,26].

Terms	Definition
Privacy	“Privacy” refers to information about a living individual that falls under any of the following items.
	a. Information that can identify an individual through name, social security number, images, etc. b. Information that can be easily identified by combining with other information, even if the information alone cannot identify a specific individual. In this case, whether or not it can be easily combined must reasonably consider the time, cost, and technology required to identify the individual, including the availability of other information.
PIA	Privacy impact assessment (PIA) refers to an assessment to analyze risk factors and derive improvements when there is a concern that the privacy of the information subject may be infringed due to the operation of a privacy file.
PIAF	PIA Framework
PII	Personal Identifiable Information

### 3.2. Comparison of PIA procedures

We examined PIAFs in the Netherlands, Canada, and South Korea and compared their implementation procedures.

#### 3.2.1. *Netherlands’s PIA procedure.*

**Fig 1:**
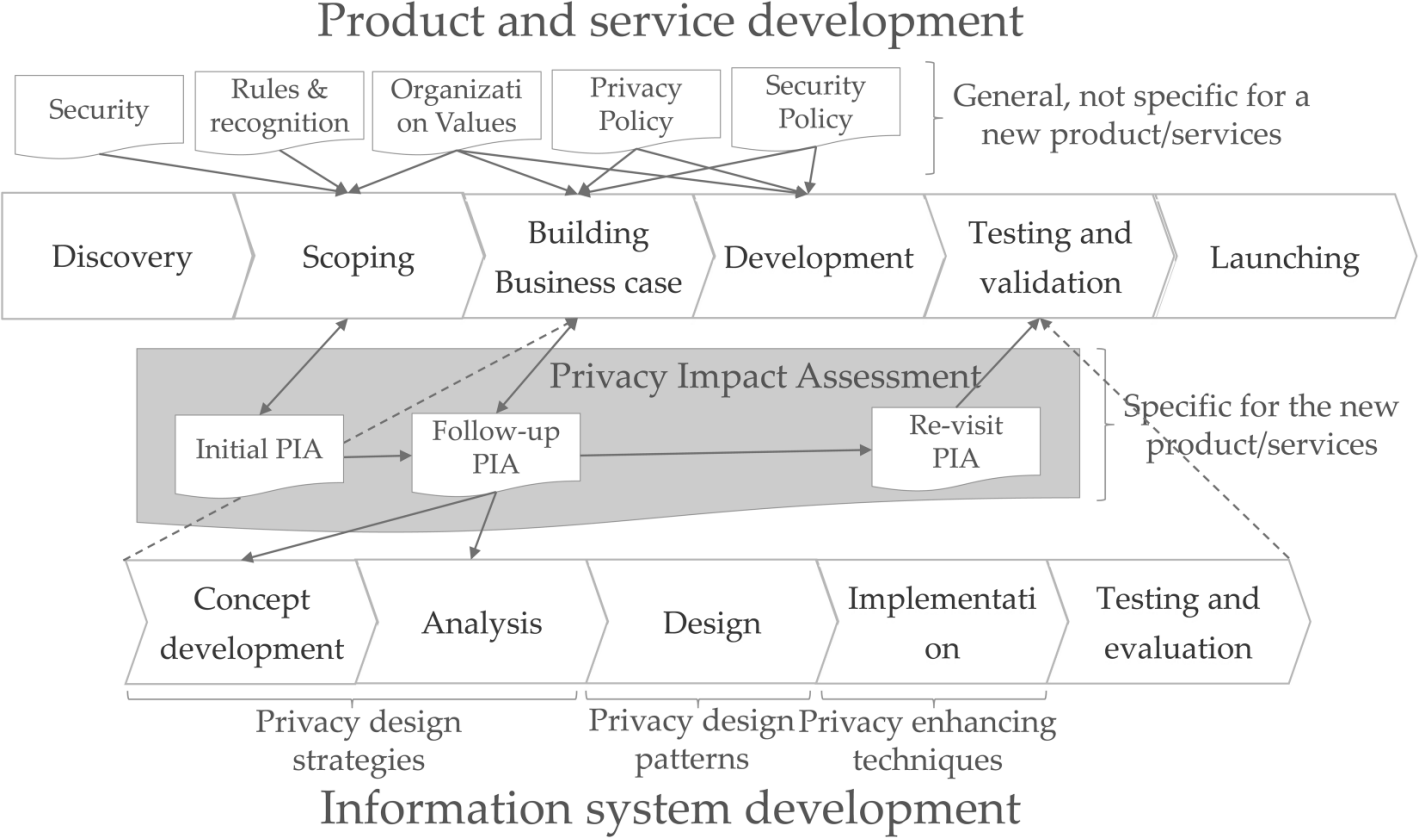
PIA in relation to product and system development in the Netherlands [8].

In the Netherlands, a PIA comprises three stages, as shown in Fig 1 [8]: initial, follow-up, and revisit PIAs. Before conducting a PIA, evaluators must be provided with information about the product and service development process and the information system development process.

#### 3.2.2. *Canada’s PIA procedure.*

**Fig 2:**
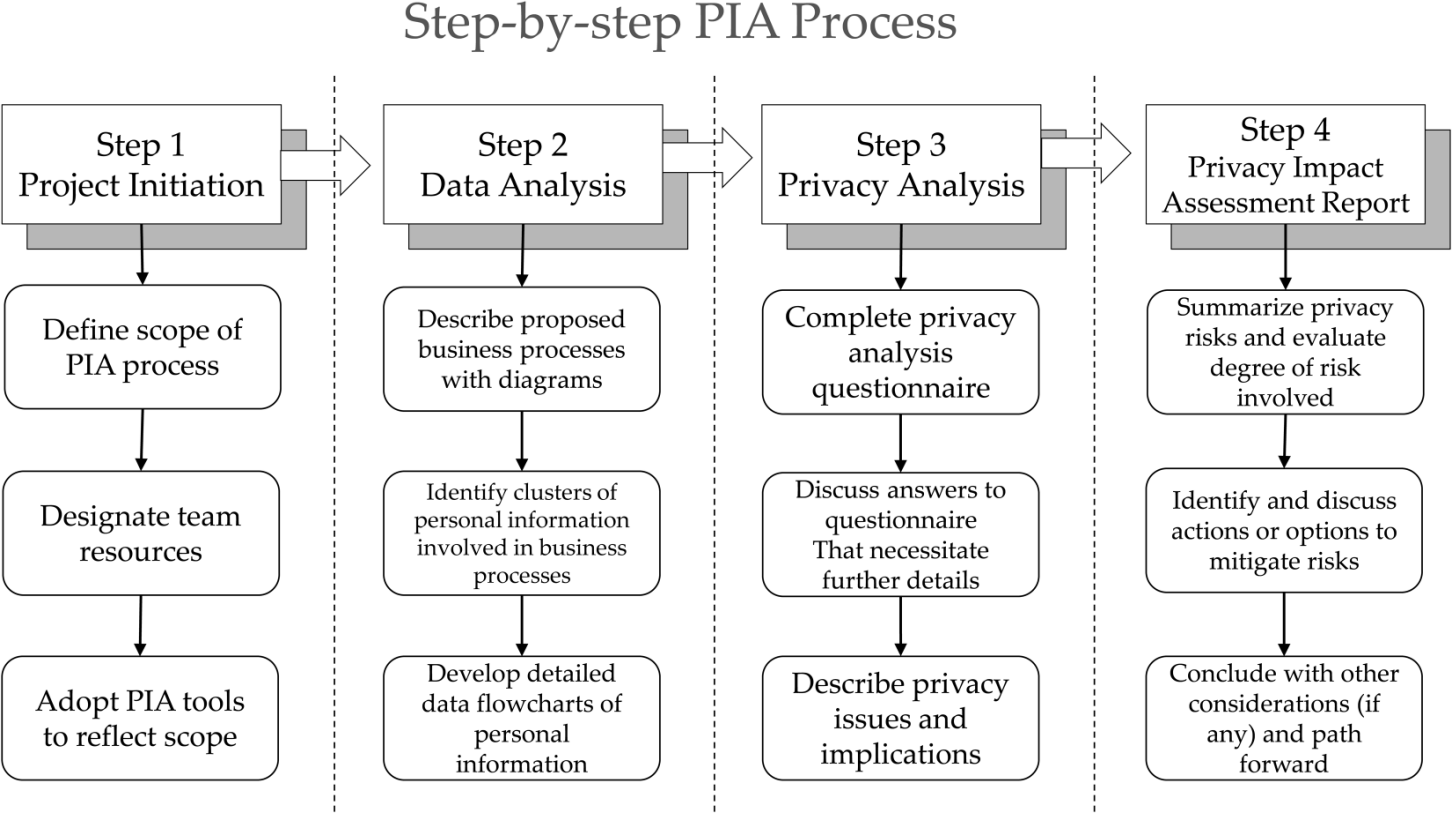
Step-by-step PIA process used in Canada [27].

In Canada, a PIA procedure comprises four stages, as shown in Fig 2 [27]: project initiation, data analysis, privacy analysis, and PIA report. Fig 2 shows the detailed tasks at each stage. In Step 3 of the privacy analysis stage, measures to mitigate or avoid negative impacts are devised through the questionnaire analysis process.

#### 3.2.3. *Korea’s PIA procedure.*

**Fig 3:**
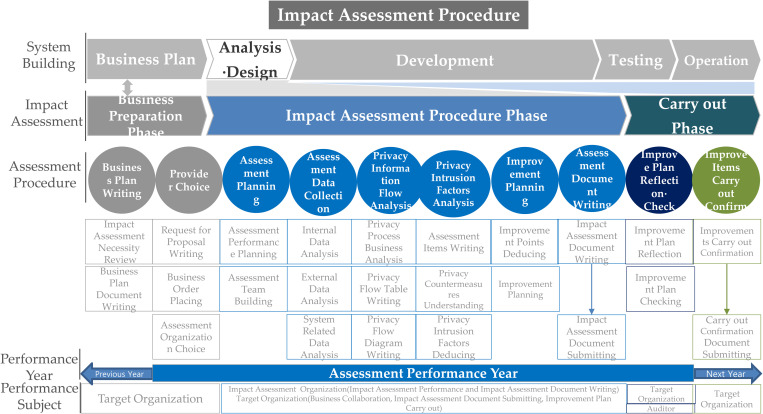
PIAF in Korea [5].

In Korea, the PIAF comprises three stages, as shown in Fig 3 [5]: preparation, execution, and implementation (or carry-out). The preparation stage comprises two sub-procedures: drafting a business plan and selecting operators. The execution stage is divided into six sub-procedures: establishing an assessment plan, collecting assessment data, analyzing privacy flow, analyzing privacy intrusion factors, developing an improvement plan, and preparing a PIA report. The implementation stage is divided into two sub-procedures: reflecting and checking the improvement plan and confirming the implementation of improvement measures, with each sub-procedure further subdivided into subprocesses.

In addition, the preparation stage is implemented a year before the assessment, the execution stage is implemented in the assessment year, and the implementation stage is implemented in the year following the assessment. This schedule and the responsible parties are specified in considerable detail.

#### 3.2.4. *Comparison of PIA procedures.*

The PIA procedure in the Netherlands is linked to the product and service development process and the information system development process. The PIA procedure in Canada consists of four stages, with each stage subdivided into three subprocesses.

After comparing the PIA procedures in Korea and overseas, the two most significant differences are the review of the necessity of a PIA and the inspection of improvement measure implementation. In overseas jurisdictions, organizations voluntarily perform a preliminary personal data impact assessment to review the necessity. However, in Korea, this requirement is mandated by the Enforcement Decree of the Privacy Protection Act and the PIA Implementation Guide.

While the inspection of improvement measure implementation is not mentioned in Europe and other regions, it is performed in Korea by a designated PIA institution or an information system audit firm in the year following the PIA. It seems necessary for other countries to define the inspection of improvement measure implementation and the PIA procedure in more detail, as is done in Korea.

### 3.3. Comparison of PIA standards

We investigated the international standard PIMS and Korea’s PIA criteria and compared them in detail for each item.

#### 3.3.1. *Criteria items in international PIMS standards.*

Table 2 shows the composition of the areas and fields of Annex A, which is a control item among the certification standards of ISO/IEC 27701:2019, an international standard for PIMSs [26]. Only 5 ISO/IEC 27001:2013 has an information security policy as a major category certification standard, but it is not shown in Table 2 for convenience.

**Table 2:** Organization of ISO/IEC 27701:2019 control items [26].

Certification Area	Middle Class Certification Standard
5. PIMS-specific requirements related to ISO/IEC 27001:2013	General
	Context of the organization
	Leadership
	Planning
	Support
	Operation
	Performance assessment
	Improvement
6. PIMS-specific guidance related to ISO/IEC 27002:2013	General
	Information security policies
	Organization of information security
	Human resource security
	Asset management
	Access control
	Cryptography
	Physical and environmental security
	Operations security
	Communications security
	System acquisition, development and maintenance Supplier relationships Information security incident management Information security viewpoints of business continuity management Compliance
7. Additional ISO/IEC 27002:2013 guidance for PII controllers	General
	Conditions for collection and processing
	Obligations to PII principals
	Privacy-by-design and privacy by default PII sharing, transfer, and disclosure

#### 3.3.2. *Standard PIA items in Korea.*

In Korea, the PIA process comprises assessment areas and items, as shown in Table 3 [4,5]. In particular, it comprises 5 assessment areas, 25 assessment fields, and 85 assessment items.

**Table 3:** Composition of PIA areas and fields [4, S3].

Assessment Area	Assessment Field	Detailed Field
1. Privacy protection management system of the target institution	1. Privacy Protection Organization	Designation of privacy protection manager and performance of role
	2. Privacy Protection Plan	Establishment of internal management plan
		Establishment of annual plan for privacy protection
	3. Privacy intrusion response	Information on how to report intrusion incidents
		Leakage incident response
	4. Guarantee of information subject rights	Establishment of data subject rights guarantee procedures and method guidance
2. Privacy protection management system of the target system	5. Privacy transactor management	Designation, management and supervision of privacy transactors
	6. Privacy file management	Privacy file ledger management
		Register privacy file
	7. Privacy policy	Disclosure and creation of privacy processing policy
3. Protection measures at each stage of privacy processing	8. Collection	Appropriateness of privacy collection
		Appropriateness of method of obtaining consent
	9. Retention	Calculation of retention period
	10. Use provision	Appropriateness of providing privacy
		Restrictions on use provision for purposes other than those intended Ensure safety when providing
	11. Consignment	Disclosure of consignment facts
		Consignment contract
		Trustee management supervision
	12. Destruction	Develop a destruction plan
		Establishment of separate storage plan
		Preparation of destruction ledger
4 Technical protection measures of the system	13. Access right management	Account management, authentication management, permission management
	14. Access control	Access control measures
		Internet homepage protection measures
		Protection measures for business mobile devices
	15. Encryption of privacy	Encryption at rest, encryption at transmission
	16. Storage and inspection of access records	Access record storage, inspection, storage, and backup
	17. Prevention of malicious programs, etc.	Vaccine installation and operation
		Apply security update
	18. Physical access prevention	Establishment of access control procedures
		Establishment of export–import control procedures
	19. Destruction of privacy	Safe destruction
	20. Other technical protection measures	Development environment control
		Privacy processing screen security
		Protective measures when printing
	21. Protection of privacy processing area	Designation of protected area
5. Privacy protection when using specific IT technology	22. CCTV	Collect opinions and provide installation guidance when installing CCTV
		Restriction of CCTV use, entrustment of installation and management
	23. RFID	RFID User Guide
		Attaching and removing RFID tags
	24. Bio information	Protective measures when storing original information
	25. Location information	Consent to collection of personal location information, instructions upon provision

- 1. The privacy protection management system of the target organization area includes 9 assessment items.
- 2. The privacy protection management system of the target system area includes 6 assessment items.
- 3. The protection measures at each stage of the privacy processing area include 21 assessment items.
- 4. The technical protection measures of the target system area include 33 assessment items.
- 5. The area of privacy protection when using specific IT technology comprises 16 assessment items.

#### 3.3.3. *Comparison of assessment areas between ISO/IEC 27701 and Korea’s PIA.*

Table 4 summarizes the results of comparing the content based on the assessment field between ISO/IEC 27701:2019, an international standard for privacy protection, and Korea’s PIA. For the 25 assessment areas of the Korean PIA, 5 of the assessment areas of ISO/IEC 27701:2019 match, 11 partially match, and 9 do not match. In particular, the area of privacy protection did not match when using specific IT technology.

**Table 4:** Comparison of assessment areas between ISO/IEC 27701 and Korean PIA [5,26].

Assessment Area	Assessment Field (PIA)	Mapping	Assessment Field (ISO/IEC 27701)
1. Privacy protection management system of the target institution	1. Privacy Protection Organization	○	Requirements: Role, accountability and authority for the organization
	2. Privacy Protection Plan	△	Requirements: General
	3. Privacy intrusion response	X	
	4. Guarantee of information subject rights	△	Control Items: Handling requests
2. Privacy protection management system of the target system	5. Privacy transactor management	X	
	6. Privacy file management	X	
	7. Privacy policy	X	
3. Protection measures for each stage of privacy processing	8. Collection	△	Control Items: Limit collection
	9. Retention	○	Control Items: Retention
	10. Use provision	△	Control Items: PII sharing, transfer, and disclosure
	11. Consignment	△	Control Items: PII controllers’ obligations to inform third parties
	12. Destruction	△	Control Items: Disposal
4. Technical protection measures of the system	13. Access rights management	○	Control Items: User access management
	14. Access control	△	Control Items: System and application access control
	15. Encryption of privacy	△	Control Items: Cryptographic controls
	16. Storage and inspection of access records	△	Control Items: Logging and monitoring
	17. Prevention of malicious programs, etc.	△	Control Items: Protection from malware
	18. Physical access prevention	○	Control Items: Physical entry controls, Management of removable media
	19. Destruction of privacy	X	
	20. Other technical protection measures	△	Control Items: Protection of test data, Clear desk and clear screen policy
	21. Protection of privacy processing	○	Control Items: Protecting against external and environmental threats, Verify, review and evaluate information security continuity, Information backup
5. Privacy protection when using specific IT technology	22. CCTV	X	
	23. RFID	X	
	24. Bio information	X	
	25. Location information	X	

※ Legend: ○: Exist; △: Exist partially; X: Does not exist at mapping column.

Compared to ISO/IEC 27701, Korea’s PIA includes additional assessment areas such as privacy protection when using specific IT technologies (e.g., CCTV, biometrics, RFID), which are not explicitly addressed in the ISO standard. This reflects Korea’s deliberate effort to operationalize privacy protections by directly linking assessment items to the technologies widely deployed in real-world information systems, thereby enabling more actionable and implementation-focused evaluations.

## 4. Case study

We conducted PIA projects on information systems built in Korean public institutions. Using these projects, we identified privacy infringement factors and proposed improvement measures for each identified factor. We calculated the frequency of each item in the PIA results that were either partially or not implemented. For items with a frequency higher than the average, we identified intrusion factors and proposed improvement measures. Table 5 summarizes the public institutions and information systems subject to PIAs.

**Table 5:** Target public institutions and related information systems [S1].

No.	Category	Target public institutions	Target information systems	Number
1	corporation	law, pension	legal system, pension system	3
2	finance	bank	bank system	1
3	public corporation	public corporation	card issuing system	1
4	local government	city, city library	local tax system, consumer information system, library management system	3
5	central administrative department	procurement service, administrative autonomy, customs service, ministry of food and drug safety	procurement system, service system, education system, information network, safety information network, non-tax system	7
6	school	college	integrated information system	1
7	court	supreme court	service system	1
8	committee	human rights commission	civil complaint system	1
	**Sum**			**18**

### 4.1. *PIA item assessment results*

Considering the scale, impact, and other factors, we limited the analysis to five of the systems summarized in Table 5. Furthermore, we excluded privacy protection areas utilizing specific IT technologies that have not yet been applied. The assessment was performed in four areas: the privacy protection management system of the target institution, the privacy protection management system of the target system, protection measures at each stage of privacy processing, and technical protection measures of the system.

Among 18 PIA reports conducted on public systems, five systems were selected for detailed analysis based on their completeness, system size, and impact scope. Items with three or more instances of partial or non-implementation were selected as candidates for intrusion factor analysis (see Table 6).

**Table 6:** List of items subject to privacy intrusion factors [S2].

Area	Field	Assessment Item	Frequency
2. Privacy protection management system of the target system	5. Privacy transactor management	2.1.1	4
		2.1.2	4
3. Protection measures for each stage of privacy processing	8. Collection	3.1.3	3
	9. Retention	3.2.1	3
	11. Consignment	3.4.2	3
4. Technical protection measures of the system	13. Access rights management	4.1.1	5
		4.1.4	5
		4.1.6	4
		4.1.7	3
		4.1.8	5
	16. Storage and inspection of access records	4.4.1	5
		4.4.2	3
	20. Other technical protection measures	4.8.2	4

The frequency of each item that was partially or not implemented was calculated. The results are summarized in Table 6. To ensure the general applicability of the case study, items with a frequency of 3 or more were selected as potentially infringing on privacy. Because the privacy protection management system area of the target institution and the privacy protection management system area of the target system had a frequency of less than 2, they were excluded from the analysis. As summarized in Table 6, 13 items with a frequency of 3 or more are subject to privacy infringement factor identification.

By aggregating the frequency of assessment items by area, as shown in Table 6, more distinct results can be derived. The total for the privacy protection management system area of the target system is eight, and the area for protection measures at each stage of privacy processing is nine. However, the total for the technical protection measures area of the system is 34. This indicates that the area of technical protection measures for the system urgently needs improvement.

To ensure the reliability of our findings and the practical effectiveness of the proposed improvement measures, we incorporated a quantitative analysis of five real-world PIA cases. We calculated the frequency of items that were partially or not implemented across these cases and used a threshold of three or more occurrences to identify high-priority risk items. These frequencies, summarized in Table 6, serve as empirical evidence to support the selection of intrusion factors and validate the applicability of the countermeasures proposed in this study.

We selected a threshold of three or more occurrences for identifying high-priority items based on the average non-implementation frequency across five case studies. This approach balances specificity and sensitivity in identifying systemic vulnerabilities. Setting a lower threshold (e.g., 2) would have increased the number of items considered but risked including less critical or case-specific issues, while a higher threshold (e.g., 4) would have excluded several recurrent issues that, while less frequent, still pose significant privacy risks.

### 4.2. Derivation of privacy intrusion factors

To identify intrusion factors, we selected items that were either partially implemented or not implemented at all, as shown in Table 6. A threshold of three or more occurrences was set to filter high-priority risks. Each selected item was examined through the lens of its assessment criteria, and specific causes of privacy intrusion were derived based on the nature of non-compliance. The risk levels were qualitatively assessed, and improvement measures were proposed according to the impact and urgency of the identified risks.

**Fig 4:**
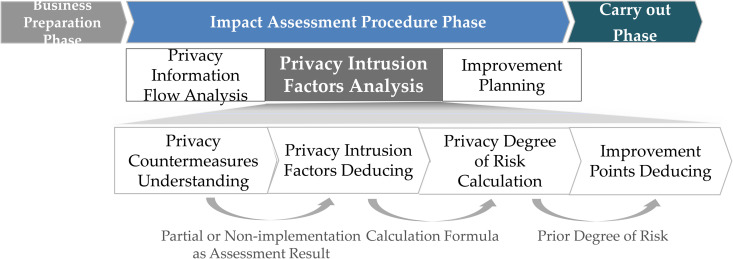
Process of analyzing privacy intrusion factors within the PIAF in Korea.

Fig 4 illustrates the process of analyzing privacy intrusion factors within the PIAF of South Korea. For items that are partially or not implemented based on the assessment results, the assessment criteria are analyzed. Through this process, privacy intrusion factors are identified, and the risk level is assessed. The process involves identifying improvement measures for each intrusion factor in descending order of risk level.

The privacy intrusion factors for the 13 items are identified by assessment area as follows:

**Item 2.1.1:** “Are you planning to designate privacy transactors so that the minimum number of people process privacy within the minimum scope required for business?” **Intrusion Factors:** Unnecessary access to privacy processing systems by personnel not required for business can increase the risk of privacy misuse and leakage incidents. Furthermore, if non-privacy transactors access the privacy processing system, quick cause tracing can be delayed in the event of privacy leakage.

**Item 2.1.2:** “Are you planning to assign roles and responsibilities to privacy transactors, provide them with privacy protection training, and have them sign privacy protection agreements for management and supervision purposes?” **Intrusion Factors:** The misuse of privacy by privacy transactors can increase the risk of infringement of data subjects’ rights and privacy leakage incidents.

**Item 3.1.3:** “Are you collecting resident registration numbers only if there is a legal basis, and are you planning to allow membership registration on your website without using resident registration numbers?” **Intrusion Factors:** Collecting resident registration numbers without a legal basis may violate the Privacy Protection Act. Moreover, there is a possibility of exposure or leakage of resident registration numbers, which are the most sensitive information, on the website.

**Item 3.2.1:** “Are you planning to calculate the retention period of privacy based on legal standards and the minimum period consistent with the purpose of retention?” **Intrusion Factors:** Retaining privacy beyond the necessary period can infringe on the rights of data subjects and increase the risk of privacy leakage incidents.

**Item 3.4.2:** “Are you planning to create documents that include responsibilities for privacy management when outsourcing tasks related to privacy processing?” **Intrusion Factors:** In the event of a privacy incident, unclear responsibility can result in disputes over indemnity between the consignor and consignee. Moreover, insufficient privacy protection standards for consignees can make the consignor’s management and supervision of the consignee ineffective.

Privacy intrusion factors in the area of technical protection measures of the system are listed in Table 7.

**Table 7:** Privacy intrusion factors in the area of system technical protection measures.

Assessment Items	Intrusion Factors
4.1.1 Are you planning to grant individual accounts to each privacy transactor to ensure traceability of responsibility?	✓ In the event of a privacy leak or exposure incident, prompt tracking of the cause through public or multiple accounts may be delayed due to confusion.
4.1.4 Do privacy transactors and managers who process large amounts of privacy or sensitive privacy plan to apply enhanced authentication methods?	✓ The risk of privacy leakage or exposure may increase due to illegal access of the privacy manager account to the privacy processing system due to cyberattacks, including SQL injection by external hackers.
4.1.6 To prevent illegal access and intrusion incidents to the privacy processing system, do you plan to automatically block access to the system if the privacy transactor does not process work for more than a certain period of time?	✓ The risk of privacy leakage or exposure may increase due to the theft of privacy transactor rights in the privacy processing system by cyberattacks, including Cookie Replay Attack and Session Hijacking, by external hackers.
4.1.7 To prevent abnormal access to the privacy processing system, are you planning to apply protection measures such as account locking, simultaneous access restrictions, and manager login notification when inactive for a long period of time?	✓ The risk of privacy being leaked, altered, or damaged may increase due to the theft of privacy transactor authority in the privacy processing system by cyberattacks, including Account Stealing and Using and Session Hijacking by external hackers.
4.1.8 If the privacy transactor changes, do you plan to change or cancel access to the privacy processing system without delay?	✓ The risk of privacy leakage or exposure may increase if a malicious privacy transactor accesses a previous privacy processing system without legitimate authority.
4.4.1 Are you planning to record all necessary information, such as identifier, access date and time, information indicating the location of access, and tasks performed, in the access records of the privacy processing system?	✓ In the event of a privacy leak or exposure incident, rapid cause tracking may be delayed due to a lack of information on the actions of the privacy transactor.
4.4.2 Do you plan to regularly check the access records of the privacy processing system in order to respond to leaks, alterations, and damage to privacy?	✓ In the event of a privacy leakage or exposure incident due to a late discovery of privacy leakage, alteration, or damage in the privacy processing system or symptoms, prompt tracking of the cause may be delayed.
4.8.2 In order to prevent leakage or exposure of privacy through privacy processing screens of privacy transactors and information subjects, privacy masking, restrictions on the right mouse button of the web browser, temporary file and cache control, and protection of important information such as card numbers are provided. Are you planning to apply protection measures such as preventing copying, screen capture, and keyboard hacking?	✓ The risk of privacy leakage or exposure may increase due to cyberattacks, including Session Hijacking, Arbitrary Modification of Source Code, and Keyboard Hacking by external hackers, and arbitrary manipulation of the privacy processing system by malicious privacy transactors.

### 4.3. Summary of privacy intrusion factors

**Fig 5:**
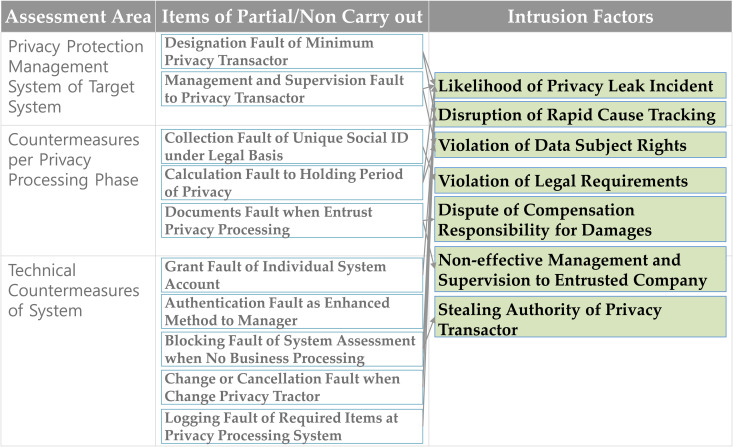
Summary of privacy intrusion factors.

The privacy intrusion factors have been categorized by assessment area and are illustrated in Fig 5. In Fig 5, the types of intrusion factors resulting from defective items are summarized into seven categories. The representative intrusion cases include the possibility of privacy leakage incidents, hindering prompt cause tracing in the event of a privacy leakage incident, and unauthorized use of the authority of privacy transactors or managers.

### 4.4. Improvement measures of privacy intrusion factors

The improvement measures for each of the 13 intrusion factors identified in the PIA are summarized in Tables 8–10 as protection measures for preventing these factors.

**Table 8:** Privacy intrusion factors and improvement points in the privacy protection management system area of the target system.

Intrusion factors	Improvement measures
The risk of misuse and leakage of privacy may increase as unnecessary personnel access the privacy processing system for work purposes In the event of a privacy leak incident due to access to the privacy processing system by personnel other than the privacy transactor, prompt tracking of the cause may be delayed	Designate privacy transactors and create a list to manage and supervise privacy so that the minimum number of people handles privacy
When processing privacy by the privacy transactor, the risk of violation of the rights of the information subject or privacy leakage may increase due to misuse of privacy	Manage and supervise assigning roles and responsibilities to privacy transactors, providing privacy protection training, and requiring security and privacy protection pledges, etc.

**Table 9:** Privacy intrusion factors and improvement points in the area of protection measures at each privacy processing stage.

Intrusion factors	Improvement measures
Violating the Privacy Protection Act by collecting resident registration numbers without legal basis There is a possibility of the most sensitive resident registration number being leaked or exposed on internet homepages	Resident registration numbers are collected only when there is a legal basis, both online for privacy processing systems and offline for paper documents Design and implement a target system to prohibit the collection of resident registration numbers when registering as a member on the Internet homepage
Retaining privacy longer than necessary may increase the risk of violating the rights of the information subject and increasing the risk of privacy leakage	Calculate the retention period of privacy below the minimum period that meets the provisions of laws and regulations related to privacy protection or the purpose of retention If there is privacy whose retention period has expired, it is destroyed both online in the privacy processing system and offline in paper documents
In the event of a privacy accident, problems may arise between the consignor and trustee over liability for damages due to unclear responsibility Management supervision of trustees by trustees may be ineffective due to insufficient privacy protection standards for trustees	When entrusting work related to privacy processing, prepare a document containing responsibilities for privacy management and monitor its implementation

**Table 10:** Privacy intrusion factors and improvement points in the area of system technical protection measures.

Intrusion factors	Improvement measures
In the event of a privacy leak or exposure incident, prompt tracking of the cause through public or multiple accounts may be delayed due to confusion	Design and implement a privacy processing system to provide individual accounts for each privacy transactor
The risk of privacy leakage or exposure may increase due to illegal access of the privacy manager account to the privacy processing system due to cyberattacks, including SQL injection by external hackers	Design and implement a privacy processing system to apply enhanced 2-factor authentication methods such as joint certificates and IP addresses to privacy transactors and managers who handle large amounts of privacy
The risk of privacy leakage or exposure may increase due to the theft of privacy transactor rights in the privacy processing system by cyberattacks, including Cookie Replay Attack and Session Hijacking, by external hackers	Design and implement a privacy processing system so that if the privacy transactor does not process work for more than a certain period of time, access to the system is automatically blocked
The risk of privacy being leaked, altered, or damaged may increase due to the theft of privacy transactor rights in the privacy processing system by cyberattacks, including account stealing and session hi-jacking by external hackers	Design and implement a privacy processing system to lock the account and limit simultaneous access when the privacy transactor is not connected to the privacy processing system for a long period of time Design and implement protection measures such as mobile phone messages and e-mail notifications when the privacy manager logs into the privacy processing system
The risk of privacy leakage or exposure may increase if a malicious privacy transactor accesses a previous privacy processing system without legitimate authority	Design and implement a privacy processing system to change or cancel access rights without delay if the privacy transactor changes
When a privacy leak or exposure incident occurs, rapid cause tracking may be delayed due to a lack of information on the actions of the privacy transactor	Design and implement a privacy processing system so that all necessary information such as identifier, access date and time, information identifying the accessor, processed information subject information, and performed tasks are recorded in the access record of the privacy processing system
In the event of a privacy leakage or exposure incident due to a late discovery of privacy leakage, alteration, or damage in the privacy processing system or symptoms, prompt tracking of the cause may be delayed	Establish a plan to check the privacy transactor’s access records to the privacy processing system at least once a month, report it to the relevant person, and implement it
The risk of privacy leakage or exposure may increase due to cyberattacks, including Session Hijacking, Arbitrary Modification of Source Code, and Keyboard Hacking by external hackers, and arbitrary manipulation of the privacy processing system by malicious privacy transactors	Design and implement a privacy processing system to mask important items when searching privacy in a list format, restrict the right mouse button of the web browser, control cache, prevent copying and screen capture of important information, and prevent keyboard hacking

In addition to audit logging and access control, data-level obfuscation techniques—such as grayscale steganography [22] and privacy protection mechanisms applied in educational systems [24]—can be leveraged to minimize the risk of unintended data exposure or leakage through visual or system-level interfaces. Furthermore, smart device privacy evaluation frameworks [23] underscore the importance of incorporating adaptive PIA assessment fields when privacy services are delivered via mobile platforms or IoT environments.

Educational service infrastructures also benefit from motivation-based models [24] that promote consistent adherence to privacy protocols among diverse user groups, reinforcing the role of user engagement in privacy management.

Medical data protection during high-risk periods, such as the Hajj season [28], and lightweight cryptographic techniques suitable for resource-constrained IoT systems [29] also support the adoption of multi-layered security architectures—complementary to the technical protection measures proposed in our study.

For instance, our proposed improvement measure related to access control (Item 4.1.1) can be realized through centralized identity and access management (IAM) systems equipped with audit trail functionality, which are already common in public sector infrastructures. Similarly, enhanced authentication (Item 4.1.4) can be implemented using two-factor authentication protocols, particularly for users with elevated privileges.

The proposed improvements (e.g., 2FA for administrators, audit logging) are consistent with ISO/IEC 27001:2013 control items such as A.9 (Access Control) and A.12 (Operations Security). These controls are widely applicable to public sector systems in other jurisdictions with similar system architectures and data processing functions.

### 4.5 Methodology for identifying intrusion factors

To systematically identify privacy intrusion factors, we adopted a multi-step analysis process using data from real-world PIAs. First, we selected five PIA cases from Korean public institutions based on system size, completeness, and scope of impact. From each PIA report, we extracted assessment results for 85 items across 5 areas. We calculated the frequency of each item that was either partially or not implemented and used a threshold of three or more occurrences to select high-priority items. Each of these items was examined based on its associated evaluation criteria to determine potential privacy risks. We then mapped these risks to specific causes—termed intrusion factors—and proposed corresponding improvement measures based on international security standards, practical feasibility, and urgency.

To justify the threshold value of three or more, we performed a frequency distribution analysis of the assessment results and observed that three represented a balanced cutoff point. Items appearing in fewer than three cases showed greater variance and were often project-specific, whereas items with three or more instances reflected repeated issues across systems. We also considered alternative thresholds and found that using two included too many marginal items, and using four excluded several meaningful but moderately frequent risk indicators.

We acknowledge that the identification and evaluation of intrusion factors in this study were based on expert judgment, which may introduce subjectivity. To address this limitation, future research will aim to incorporate structured stakeholder-based evaluations, such as Delphi techniques or quantitative surveys, to validate the analysis and improve its objectivity.

### 4.6 Differentiation from existing methods

Unlike traditional PIA frameworks that focus primarily on compliance checklists or general risk categories, our approach emphasizes case-driven analysis. Previous studies have suggested frameworks for organizing PIA processes but often lack actionable insights derived from field data. In contrast, we base our analysis on actual PIA reports conducted in public institutions and extract item-level intrusion patterns from frequency-based performance metrics. This enables us to propose operationally feasible improvement strategies tailored to frequently occurring weaknesses, thereby bridging the gap between theoretical assessment structures and practical implementation.

## 5. Discussion

Previous studies on PIAs were analyzed. From a methodology perspective, the PIAF had been treated at a high level of major categories; thus, detailed fields and specific assessment items for impact assessment were not presented. In particular, we could not find any research that provides information about intrusion factors and improvement measures. Therefore, it was insufficient to conduct PIAs in the field by referring to the results of previous studies.

We compared the assessment areas of Korea’s PIA with those of ISO/IEC 27701. Among the PIA areas in Korea, the privacy protection management system of the target system and the protection of privacy when utilizing specific information technologies did not match with the PIA area of ISO/IEC 27701. The concept of privacy protection originated in Europe and has continued to advance, leading to the establishment of international standards. Korea also introduced the PIA system and added new assessment areas in the process of supplementing it to suit the Korean situation. The privacy protection management system area of the target system must ensure the rights of information subjects. In addition, in the area of privacy protection when using specific information, there are many privacy violation technologies in the fields of CCTV and bio information.

We executed PIA projects on integrated information systems operated by public institutions in Korea. Based on the assessment items in four areas, we selected the items that were evaluated as partially or not implemented. We used the value of three items evaluated as insufficient preparation as a threshold. For the 13 assessment items with low performance, we derived privacy instruction factors and proposed improvement measures for each factor.

In our PIA project, we performed a risk assessment for each item that was evaluated as partially or not implemented and proposed improvement measures according to the risk level. An improvement plan was established by classifying improvement measures considering risk, urgency, and budgetary circumstances. Finally, a PIA report was prepared by synthesizing all outputs from the PIA project.

Our proposed methodology enhances the completeness of privacy impact assessments by systematically identifying partially implemented items, tracing them to their root causes, and suggesting mitigation strategies grounded in both policy and technical safeguards. This approach helps reduce the likelihood of undetected vulnerabilities, which are often overlooked in traditional high-level PIA frameworks.

Our findings are also consistent with prior trust-based security models. For example, three-layered privacy protections in healthcare systems [28], steganographic techniques for data concealment [21,22], and risk mitigation strategies in smart-community IoT contexts [30] all highlight the importance of system-level accountability and multi-layered resilience against intrusions. Moreover, identity-based systems such as face recognition [31] introduce both ethical and functional privacy risks—supporting our argument for explicitly incorporating biometric and location-based items in PIA assessments.

The successful implementation of PIA recommendations often depends not only on technical safeguards but also on organizational culture and user engagement. As demonstrated in [27], fostering trust and encouraging proactive participation in privacy-protecting IT practices—particularly among professionals such as educators—is crucial. This reinforces our recommendation to assign clear privacy responsibilities and provide targeted training to reduce internal risks.

While this study offers a detailed examination of PIA practices within Korean public institutions, we acknowledge that the generalizability of the findings may be limited by the geographical and regulatory context. To address this, future research should aim for cross-national collaboration to validate and adapt the proposed methodology in diverse jurisdictions with varying PIA structures and threat landscapes.

In practice, public institutions in Korea are required to conduct PIAs during the analysis and design phases of new information systems. The systems must be designed in accordance with the identified improvement measures, and evaluators confirm during the testing phase whether these measures have been properly implemented. This procedural linkage between assessment, design, and verification reinforces the practical utility of our methodology and highlights its potential for broader application.

Our comparative analysis also highlights that procedural and legal mandates embedded in national PIA frameworks substantially affect implementation outcomes. For example, Korea’s requirement for post-assessment audits by designated institutions ensures accountability beyond the assessment phase, while the voluntary nature of PIAs in countries like the Netherlands may limit follow-up actions. Similarly, Korea’s legal enforcement of detailed technical assessment areas drives more consistent execution in practice. These differences suggest that not only the structure, but the enforceability and oversight mechanisms of a PIA framework, critically shape how privacy risks are addressed on the ground.

Despite the strengths of our data-driven methodology, this study has several limitations. First, the generalizability of our findings may be constrained by the geographic and institutional specificity of the data—PIAs from Korean public sector systems. Second, the evaluation of intrusion factors is qualitative, based on expert interpretation of assessment criteria and institutional contexts. Third, although we present improvement measures aligned with international standards, we did not conduct post-implementation follow-up to quantify actual improvements in system security or privacy outcomes. Future research should expand the dataset across jurisdictions and include longitudinal studies to assess the impact of implemented countermeasures over time.

In future research, we plan to complement expert-based assessments with input from a broader set of stakeholders. By utilizing methods such as Delphi panels or structured questionnaires targeting system users, developers, and privacy officers, we aim to enhance the reliability and generalizability of intrusion factor evaluation

## 6. Conclusions

In our analysis of the PIAF cases, we found that the Netherlands has a three-step process, whereas Canada has a four-step process with three subprocesses for each step. The Korean PIAF, which is organized into three phases, defines the process in detail and specifically mentions when and by whom it should be performed. To compare from an implementation perspective beyond the legal and institutional perspectives of PIAs, we compared the procedures in ISO/IEC 29134 and Korea’s PIA Implementation Guide. The ISO/IEC 29134 table of contents was outlined at a high level in terms of taxonomy.

Consequently, we could not find any data or studies that could be used as a reference to analyze intrusion factors or suggest improvement plans according to the assessment results. We believe that this may be due to the limitations of the data we have obtained. In contrast, we believe that overseas researchers may not have acquired sufficient data on Korea’s PIAs. We expect that this study will provide a reference for other researchers by presenting the detailed items presented in Korea’s PIAF.

The results of the PIA projects executed by public organizations in Korea, which were discussed in Chapter 5, are likely to be similar in other countries. We believe that the hardware architecture and software systems of integrated information systems operating overseas are similar to those in Korea. Various cyberattacks such as session hijacking and cookie replay attacks targeting information systems will also apply to domestic and foreign information systems. As revealed in the case of Korea, items related to the technical protection measures of the system are considered important issues in the implementation of PIAFs. In particular, it will help prevent incidents of violation of the rights of information subjects when operating information systems and during privacy leakage from inside and outside the organization due to various cyberattacks. We also believe that this will contribute to the prevention of secondary damage caused by cyberattacks such as voice phishing.

We expect that our research on intrusion factors and improvement measures presented in depth at the level of assessment items will serve as a reference for public and private sector organizations around the world.

## Supporting information

S1 Table Intrusion-factors, table. list of items subject to privacy intrusion factors. (DOC)

S2 Table Information-infringement. table. list of disclosed items related to personal information infringement. (DOC)

S3 Table Infringement-factors. table. comparative table of infringement factors: author’s assessment vs. publicly available data. (DOC)
