# Supplementary material for: Common intrusion factors and improvement measures based on case study of privacy impact assessment
Source: PLoS One. 2025 Aug 25;20(8):e0328180. doi: 10.1371/journal.pone.0328180 (PMC12377571; doi:10.1371/journal.pone.0328180)
Supplement: S1 Table — (DOC) [file pone.0328180.s001.doc]

We conducted PIA projects on information systems built in Korean public institutions. Among 18 PIA reports conducted on public systems, five systems were selected for detailed analysis based on their completeness, system size, and impact scope. Items with three or more instances of partial or non-implementation were selected as candidates for intrusion factor analysis. The frequency of each item that was partially or not implemented was calculated. The results are summarized in Table 1.

**Table . List of items subject to privacy intrusion factors**

| **Area** | **Field** | **Assessment Item** | **Frequency** |
| --- | --- | --- | --- |
| 1. Privacy protection management system of the target institution | 1. Privacy Protection Organization | 1.1.1  1.1.2 | 1  1 |
|  | 2 Privacy Protection Plan | 1.2.1 | 1 |
|  |  | 1.2.3 | 1 |
|  | 3. Privacy intrusion response | 1.3.2 | 1 |
|  | 4. Guarantee of information subject rights | 1.4.1 | 1 |
|  |  | 1.4.2 | 1 |
| 2. Privacy protection management system of the target system | 5. Privacy transactor management  6. Privacy file management | 2.1.1  2.1.2  2.2.1 | 4  4  2 |
|  |  | 2.2.2 | 1 |
|  | 7. Privacy policy | 2.3.1  2.3.2 | 2  1 |
|  |  |  |  |
| 3. Protection measures at each stage of privacy processing | 8. Collection | 3.1.1  3.1.3  3.1.4  3.1.5  3.1.6  3.1.7 | 2  2  2  1  2  1 |
|  | 9. Retention | 3.2.1 | 3 |
|  | 10. Use provision | 3.3.1  3.3.2  3.3.5  3.3.6  3.3.7 | 2  1  2  2  4 |
|  | 11. Consignment | 3.4.2  3.4.3 | 3  1 |
|  | 12. Destruction | 3.5.1  3.5.3 | 2  1 |
| 4. Technical protection measures of the system | 13. Access right management | 4.1.1  4.1.3  4.1.4  4.1.6  4.1.7  4.1.8  4.1.9  4.1.10 | 5  1  4  3  3  4  2  2 |
|  | 14. Access control | 4.2.3  4.2.4  4.2.5 | 1  2  3 |
|  | 15. Encryption of privacy | 4.3.3 | 2 |
|  | 16. Storage and inspection of access record | 4.4.1  4.4.2  4.4.3 | 5  3  2 |
|  | 17. Prevention of malicious programs, etc. | 4.5.1  4.5.2 | 1  3 |
|  | 18. Physical access prevention | 4.6.1  4.6.2 | 2  1 |
|  | 19. Destruction of privacy | 4.7.1 | 2 |
|  | 20. Other technical protection measures | 4.8.1  4.8.2  4.8.3 | 1  5  2 |
|  | 21. Protection of privacy processing area | 4.9.1  4.9.2 | 2  2 |
