# Supplementary material for: Common intrusion factors and improvement measures based on case study of privacy impact assessment
Source: PLoS One. 2025 Aug 25;20(8):e0328180. doi: 10.1371/journal.pone.0328180 (PMC12377571; doi:10.1371/journal.pone.0328180)
Supplement: S2 Table — (DOC) [file pone.0328180.s002.doc]

The target items with a high likelihood of infringing on personal information, as identified in the analysis of 20 publicly available personal information impact assessment summaries, were categorized by impact assessment area.

**Table . List of Disclosed Items Related to Personal Information Infringement**

| **Assessment Area** | **Assessment Field** | | **Assessment Item** | **Frequency** |
| --- | --- | --- | --- | --- |
| 1. Privacy protection management system of the target institution | 2. Privacy Protection Plan  4. Guarantee of information subject rights | | 1.2.1  1.4.1 | 3  2 |
| 2 Privacy protection management system of the target system | 6. Privacy file management  7. Privacy policy | | 2.2.1  2.2.2  2.3.1  2.3.2 | 2  3  2  3 |
| 3 Protection measures at each stage of privacy processing | 8. Collection  9. Retention  11. Consignment  12. Destruction | | 3.1.1  3.1.4  3.1.6  3.1.7  3.2.1  3.4.1  3.5.1  3.5.3 | 2  2  1  1  3  3  3  1 |
| 4. Technical protection measures of the system | | 13. Access right management  14. Access control  15. Encryption of privacy  16. Storage and inspection of access record  18. Physical access prevention.  19. Destruction of privacy  20Other technical protection measures | 4.1.4  4.1.5  4.1.7  4.1.8  4.2.2  4.3.1  4.3.2  4.3.3  4.4.1  4.4.2  4.4.3  4.6.2  4.7.1  4.8.2  4.8.3 | 2  5  4  2  1  3  4  1  1  1  1  1  1  4  1 |
