# Supplementary material for: Common intrusion factors and improvement measures based on case study of privacy impact assessment
Source: PLoS One. 2025 Aug 25;20(8):e0328180. doi: 10.1371/journal.pone.0328180 (PMC12377571; doi:10.1371/journal.pone.0328180)
Supplement: S3 Table — (DOC) [file pone.0328180.s003.doc]

A comparison was made between the number of infringement factors identified in the author’s personal information impact assessment and those found in publicly available personal information impact assessment summaries. The results suggest that the author’s assessment was conducted more thoroughly and accurately than other existing assessments.

**Table. Comparative Table of Infringement Factors: Author’s Assessment vs. Publicly Available Data**

| **Assessment Area** | **Assessment Field** | **Assessment Item** | match | **publicly available**  **Assessment item** |
| --- | --- | --- | --- | --- |
| 2. Privacy protection management system of the target system | 5. Privacy transactor management  6. Privacy file management | 2.1.1  2.1.2  2.2.1 | X  X  ○ | 2.2.1 |
|  |  | 2.2.2 | ○ | 2.2.2 |
|  | 7. Privacy policy | 2.3.1  2.3.2 | ○  ○ | 2.3.1  2.3.2 |
| 3. Protection measures at each stage of privacy processing | 8. Collection | 3.1.1  3.1.3  3.1.4  3.1.5  3.1.6  3.1.7 | ○  X  ○  X  ○  ○ | 3.1.1  3.1.4  3.1.6  3.1.7 |
|  | 9. Retention | 3.2.1 | ○ | 3.2.1 |
|  | 10. Use provision | 3.3.1  3.3.2  3.3.5  3.3.6  3.3.7 | X  X  X  X  X |  |
|  | 11. Consignment | 3.4.2  3.4.3 | X  X  X | 3.4.1 |
|  | 12. Destruction | 3.5.1  3.5.3 | ○  ○ | 3.5.1  3.5.3 |
| 4. Technical protection measures of the system | 13. Access right management | 4.1.1  4.1.3  4.1.4  4.1.6  4.1.7  4.1.8  4.1.9  4.1.10 | X  X  ○  X  X  ○  ○  X  X | 4.1.4  4.1.5  4.1.7  4.1.8 |
|  | 14. Access control | 4.2.3  4.2.4  4.2.5 | X  X  X  X | 4.2.2 |
|  | 15. Encryption of privacy | 4.3.3 | X  X  ○ | 4.3.1  4.3.2  4.3.3 |
|  | 16. Storage and inspection of access record | 4.4.1  4.4.2  4.4.3 | ○  ○  ○ | 4.4.1  4.4.2  4.4.3 |
|  | 17. Prevention of malicious programs, etc. | 4.5.1  4.5.2 | X  X |  |
|  | 18. Physical access prevention | 4.6.1  4.6.2 | X  ○ | 4.6.2 |
|  | 19. Destruction of privacy | 4.7.1 | ○ | 4.7.1 |
|  | 20. Other technical protection measures | 4.8.1  4.8.2  4.8.3 | X  ○  ○ | 4.8.2  4.8.3 |
|  | 21. Protection of privacy processing area | 4.9.1  4.9.2 | X  X |  |
| Total | 52 | 47 |  | 27 |
